# Techno-Economic Prospects and Desirability of 3D Food Printing: Perspectives of Industrial Experts, Researchers and Consumers

**DOI:** 10.3390/foods9121725

**Published:** 2020-11-24

**Authors:** Siddharth Jayaprakash, Jaakko Paasi, Kyösti Pennanen, Iñigo Flores Ituarte, Martina Lille, Jouni Partanen, Nesli Sozer

**Affiliations:** 1 Department of Mechanical Engineering, Aalto University, Otakaari 4, 02150 Espoo, Finland; jouni.partanen@aalto.fi; 2 VTT Technical Research Centre of Finland Ltd., P.O. Box 1000, FI-02044 VTT Espoo, Finland; jaakko.paasi@vtt.fi (J.P.); kyosti.pennanen@vtt.fi (K.P.); martina.lille@vtt.fi (M.L.); nesli.sozer@vtt.fi (N.S.); 3 Faculty of Engineering and Natural Sciences, Tampere University, 33720 Tampere, Finland; inigo.floresituarte@tuni.fi

**Keywords:** 3D food printing, mixed methods research, expert interview, expert survey, consumer focus groups, use cases, hardware-software system, techno-economic potential, business modelling, personalized nutrition

## Abstract

3D food printing is an emerging food technology innovation that enables the personalization and on-demand production of edible products. While its academic and industrial relevance has increased over the past decade, the functional value of the technology remains largely unrealized on a commercial scale. This study aimed at updating the business outlook of 3D food printing so as to help entrepreneurs and researchers in the field to channel their research and development (R&D) activities. A three-phase mixed methods approach was utilized to gain perspectives of industrial experts, researchers, and potential consumers. Data were collected from two sets of interviews with experts, a survey with experts, and consumer focus group discussions. The results gave insights into key attributes and use cases for a 3D food printer system, including the techno-economic feasibility and consumer desirability of identified use cases. A business modelling workshop was then organized to translate these results into three refined value propositions for 3D food printing. Both the experts and consumers found personalized nutrition and convenience to be the most desirable aspects of 3D food printing. Accordingly, business models related to 3D printed snacks/meals in semi-public spaces such as fitness centers and hospitals were found to offer the highest business potential. While the technology might be mature enough at component level, the successful realization of such high-reward models however would require risk-taking during the developmental phase.

## 1. Introduction

3D printing (3DP) refers to an advanced production technology category characterized by layer-by-layer material deposition for creating personalized objects from pre-designed digital models. It has evolved significantly throughout the last three decades and 3D printers (the associated machine systems) are currently able to utilize a wide range of raw materials, including food ingredients. Until recently, the idea of automated food personalization was only associated with science fiction. 3D food printing, which integrates 3DP technology and digital gastronomy [1], has the potential to make this a reality. Even though the first 3D food printer patent was filed in 2001 [2], the technology remained under-researched until the inception of the first prototype at Cornell University in 2006 [3,4,5]. Since then, its academic relevance has increased substantially. The identified application areas of 3D food printing presently range all the way from domestic and restaurant kitchens to spaceships [6,7,8]. Moreover, the associated ingredients portfolio is expanding from naturally printable food ingredients [3] like sugar, hummus, chocolate, and cheese to healthier and more sustainable food ingredients including plant-based proteins [9,10].

Conventional food value chains, which involve centralized factory production, promote mass standardization of food products. However, this often results in over-exploitation of resources, high emissions, and food waste [11,12]. With globalization, food supply chains are increasingly becoming complex and less transparent. Shipping of pre-processed food from a centralized factory to different parts of the world not only entails a profusion of air miles, but also requires food additives to preserve the flavor and appearance. Consumer desirability of such standardized food products is gradually diminishing. 3D food printing, still in its nascent state, has the potential to address many of the challenges associated with traditional food production practices. It could make the existing food value chains more sustainable and consumer-desirable by facilitating on-demand food production, reducing food waste, and enabling automated food personalization [11,12].

**Figure 1:**
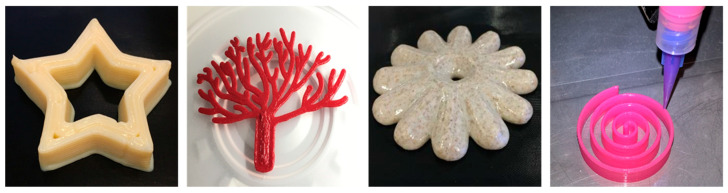
Example food structures made using 3D food printing (source: VTT Technical Research Centre of Finland).

3D food printing offers the leeway to tailor contents of food products to match an individual’s health and activity level as well as their personal taste preferences. Therefore, in addition to shape customization (Figure 1), nutritional content and texture of the food can be fine-tuned. While the potential of 3D food printing is clearly evident, its use is still limited to the academic arena, catering, and confectionery sectors [7,8]. Most existing systems are not designed for large volume applications and lack functional value propositions, well defined customer segments, and profit mechanisms. However, the scarcity of commercial success stories of 3D food printing does not point to a lack of market for the technology. BIS Research, for example, has predicted the 3D food printing market to reach $525.6 million globally by 2023 [13].

Since 3D food printing is a relatively recent innovation, there are several technological challenges to overcome before it can be adopted by the mainstream food industry [14]. For instance, matching food ingredients with suitable 3DP technologies as well as the selection and optimization of food ingredients are two major avenues that are currently being tackled [15,16]. Studies reveal that the ingredient choice for 3D food printing has a significant impact on mixing efficiency, printing speed, geometric accuracy, and compatibility with conventional post-processing techniques such as baking [1,16,17,18,19]. In addition to understanding the 3D printability of traditional food ingredients [15,18,20], there are investigations focused on the applicability of novel-alternative and healthy food ingredients for 3D food printing [9]. Despite the progress made, the functional potential of 3D food printing remains largely unrealized on a commercial level.

Researchers argue that 3D food printing will not completely replace the traditional food production process, but would diversify the food product offering [6,19]. Various application areas are identified, but the techno-economic feasibility and consumer desirability of the use cases remain unclear. Digital platforms, for example, have become an integral part of the present-day consumer experience. Previous studies have examined the possible interactions between 3DP and digital platforms [21,22], but have been mostly limited to the specialty goods sector. Identifying potentially desirable attributes of the hardware-software system for 3D food printing is expected to have a positive impact on the market acceptance of the technology. This knowledge should also help entrepreneurs and academics in the field to effectively channel their developmental activities. Additionally, a breakthrough of 3D food printing into commercial arenas may not only be dependent on the progress of technology R&D, but also on innovative business models [23,24,25].

The motivation behind this study was to update the business outlook of 3D food printing beyond general excitement in order to direct R&D efforts needed for the realization of commercial 3D food printing applications. It aimed at addressing the identified knowledge gaps through three research objectives, which are (a) gathering expert perspectives on the maturity level of 3D food printing along with the key attributes of its hardware-software system, (b) identifying use cases for 3D food printing that are techno-economically feasible as well as desirable to consumers, and (c) investigating the potential of 3D food printing in creating profitable businesses until the year 2024 by using an approach alternative to methodologies of market research, i.e., business modelling [26].

## 2. Methodology

**Figure 2:**
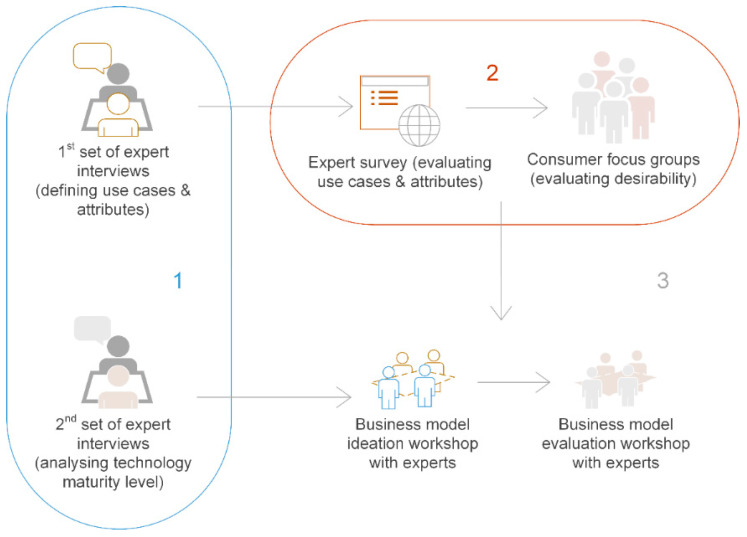
Three phases of the study, viz. (1) exploratory phase, (2) evaluation phase, and (3) development phase.

The study utilized a three-phase Mixed Methods Research (MMR) process, as illustrated in Figure 2. MMR is a subset of multimethodology [27] that systematically integrates qualitative and quantitative data as part of a study or a group of related studies [28,29]. The three associated phases are (1) exploratory phase, (2) evaluation phase, and (3) development phase, respectively. Exploratory phase included two sets of interviews with experts from within the 3D food printing value chain. While the first set of interviews aimed at gathering expert insights on use cases and potential attributes of 3D food printer systems [30,31], the second set of interviews focused on understanding the maturity level of the technology for industry-scale utilization. The second phase involved screening for the most potential use-cases utilizing an expert survey [31] and consumer focus group discussions. Here, perspectives from academia, industry, and consumers are considered for evaluating techno-economic feasibility and desirability of the use cases. In the third phase, a business modelling workshop was organized to sketch and evaluate value propositions for the selected use cases.

### 2.1. Exploratory Phase: Interviews with Experts

The first set of interviews were organized during autumn 2017, and covered topics including change drivers for 3D food printing, its hardware and software platform, key customization parameters, novel use cases, and future scenarios (Appendix A). A semi-structured and open-ended format was followed to provide enough flexibility to both the interviewer and the experts interviewed. A total of fifteen interviews with participants from Finland (10), Spain (2), Netherlands (2), and England (1) were conducted. Due to the multidisciplinary nature of the topic, a diverse interview pool was required for amassing fresh data from multiple perspectives. The participating experts represented research organizations, academia, food producers, food processing brands, food distributers, future-foresight consultancy, 3D food printing businesses, software providers, and hardware providers. While face-to-face sessions were conducted for participants from Finland, Skype was utilized as a platform for the international interviews. All the interviews were recorded (~600 min) and were later transcribed into text format. Data were visualized and analyzed using affinity diagrams [32], which facilitated organizing the ideas and opinions into categories based on their natural relationships.

A second set of semi-structured and open-ended interviews was carried-out in autumn 2018. These interviews focused on the technological feasibility of the key elements of the 3D food printing process, i.e., 3D food printing technologies, ingredients and recipes, equipment hardware and software, post-processing, and automation of printing systems. The participants either had hands-on R&D experience in the field of 3D food printing or were actively following the field with business interests. In total, 10 experts were individually interviewed from the research sector and industry in Finland, covering all the key elements of 3D food printing process. The duration of a typical interview was one hour, and the objective was to obtain a worthwhile perspective of the current feasibility of the technology, as well as its prospect feasibility within the next five years. The interviews were supplemented by findings in the literature to further define the maturity and readiness level of the specific technology or process part in question.

### 2.2. Evaluation Phase: Expert Survey and Consumer Focus Groups

#### 2.2.1. Survey Design

**Figure 3:**
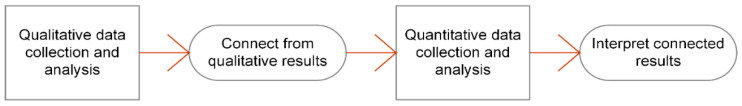
Exploratory sequential model followed as part of the survey design [33].

In order to evaluate and generalize the results of the first set of interviews, an expert survey was conducted during autumn 2017. The quantitative survey was designed following the exploratory sequential model proposed by Curry et al. [33], as shown in Figure 3. Data were gathered systematically from a pre-established target audience utilizing an online questionnaire [34]. This method was selected because of the design flexibility it allows for the researcher, convenience for the respondents, and the ease of data handling. The questionnaire was prepared in a professional electronic survey system Webropol [35], with an approximate completion time of 15 min. It consisted of multiple-choice closed-ended questions arranged into various sections, i.e., taxonomy and background information, platform for 3D food printing, use cases for 3D food printing platform, and contact information.

A 7-point Likert scale was used in the survey, with questions structured in a matrix form. The targeted experts were asked to rate (1) attributes of 3D food printing hardware, (2) attributes of 3D food printing software, (3) use-cases based on business potential, and (4) use-cases based on techno-economic feasibility. ‘1’ on the Likert scale denoted least importance/techno-economic feasibility/business potential, and ‘7’ denoted most importance/techno-economic feasibility/business potential. Additionally, an ‘I don’t know’ option was added for the participants who were unsure of how to rate. After logically structuring all the questions, the framework was tested and refined in a pilot study. It was then privately sent out to 85 experts associated with different facets of 3D food printing, with a customized invitation letter. These experts included authors of 3D food printing-related research papers, participants of previously conducted qualitative study, and representatives from 3D food printing businesses globally.

#### 2.2.2. Statistical Analysis of Survey Data

Some of the respondents constantly selected the ‘I don’t know’ option in the survey. Such responses were treated as unreliable and were removed before exporting the data to Statistical Product and Service Solutions (SPSS) software for detailed statistical analysis. Table 1 shows the number of responses (N) that were analyzed in SPSS for each of the four questions. Since the dependent variable in the questions is ordinal, non-parametric statistical analysis using a Friedman test was conducted. The test provided χ2 value (Chi-square), degrees of freedom (df), and the significance value (Asymp. Sig., *p*-value), thereby revealing any statistically significant differences between the mean ratings of related groups. While the Friedman test shows whether there are overall differences (*p* < 0.05), it doesn’t pinpoint which of the analyzed groups differ significantly from each other [36,37]. Post hoc Wilcoxon signed-rank tests were carried out on different combinations of the related groups to examine where the differences occur [38]. The test statistics gave the *Z*-scores and the corresponding Asymp. Sig. (2-sided *p*) values. These p-values were then manually compared with Bonferroni-adjusted significance level for examining the statistical significance. Bonferroni adjustment was made by dividing the initially used significance level (in this case, 0.05) with the number of tests performed [39]. The significance level was set at *p* ≤ 0.001 for all the post-hoc pair-wise comparisons.

#### 2.2.3. Consumer Focus Groups

Focus group discussions were conducted to gain insight into consumer attitude towards 3D food printing and its associated products/services. Focus groups took place in Finland and Belgium. The reasoning behind the selection of these two countries was that they do not have similar traditions in food and eating, and therefore should bring diversity to the study. There were four focus groups in Finland and three in Belgium, with 6–8 participants per group, aged between 24 and 65 years. The main criterion for the selection of participants was that they should not work or study in the fields of food production, home appliance production, and consumer/market research. The focus group discussions were conducted during spring 2018. In the discussions (Appendix B), attention was paid to participants’ spontaneous reactions to 3D food printing, their beliefs, perceptions, fears towards the technology, and potential ways of using it. The discussions also included evaluation of the potential use cases (referred to as concepts in the focus groups) that were identified in the previous research phase. The discussions were tape-recorded, and detailed notes were made throughout, which included participants’ perceptions about the potential use-cases and further development ideas. Before the discussions, all the participants provided written informed consent to take part in the study.

### 2.3. Development Phase: Business Modelling

A business model provides a holistic picture on how a firm creates and captures value. It gives answers to questions in four dimensions [26]: (1) market domain—who are the target customers and what are their needs? (2) the value proposition of the firm—what will be offered to the customers? (3) technical domain—how is the value proposition created? and (4) description of profit mechanism—how does the business model generate profit? The creation of business models forces us to simultaneously consider both the feasibility of the technology required for value creation, and the potential attractiveness of value proposals in markets for capturing value. This approach has an advantage over the typical technological feasibility or market research studies where researchers focus on either one of the subjects, neglecting the other.

Business modelling was carried out during spring 2019 by means of two separate workshops with experts (business model ideation workshop and business model evaluation workshop, as illustrated in Figure 2). Design criteria were defined beforehand to guide the ideation process, which included one criterion for each of the four dimensions of a business model. An additional criterion related to overall sustainability was also established. The results of the second set of expert interviews were utilized at this stage, on which to base the technological aspects associated with value creation. During the first workshop (business model ideation workshop), ideas for value proposition were generated by taking the findings from evaluation phase into account. These were then rendered into business model sketches that take technological feasibility aspects and market potential into account. The participants of the first workshop were the same experts who took part in the second set of interviews.

After the business model ideation workshop, three business model ideas that best demonstrated the versatility of 3D food printing were selected for further development. During the second workshop (business model evaluation workshop), initial sketches of these three selected business models were presented, evaluated, and enriched. Most of the experts that participated in the second set of interviews and the first business modelling workshop also took part in the second workshop. The evaluation was performed against the defined design criteria, technological feasibility, and business potential. The second workshop resulted in a polished set of three business models for 3D food printing.

## 3. Results

### 3.1. Exploratory Phase: Interviews with Experts

The first set of interviews carried out as part of the research helped in identifying the key change drivers that are transforming 3D food printing. As shown in Table 2, these include information and communications technology (ICT) revolution, demand for customization, the paradigm shift from centralized to on-demand food production, novel food ingredients, and dynamic markets. Interviews pointed out a broader change in eating habits, which is leading to an increased demand for personalized food products made from healthy and sustainable ingredients. In one of the personal interviews, co-founder and CEO of a 3D food printer company reiterated that “consumers and businesses are becoming aware of the over exploitation of resources in food production, and the carbon food-print/pollution associated with food processing.” Additionally, the boom in food-tech market together with the advancements in 3D printing and the role of digital platforms, cemented 3D food printing as an exciting exploitable asset in the mind of experts. However, they indicated that its economic potential is still reliant on factors such as the maturity level of the technology, where and how it is utilized, and the hardware-software system design.

#### 3.1.1. Hardware-Software System for 3D Food Printing

The key attributes of 3D food printer hardware and software that are gathered from the interviews are listed in Table 3. Software here refers to either a smart phone application or an online platform for the consumers. In general, the interviewed experts pointed towards a modular hardware-software platform that offers transparency and convenience. While the software attributes are found to be feasible considering the recent ICT developments, hardware attributes such as integrated processing system and packaging solutions would demand further R&D work. Regardless, experts recognized the importance of hardware-software system design in determining the market success of 3D food printing. In terms of customization, nutritional content, flavor, food texture, portion size, and shape were considered to be the most pertinent parameters. According to an interviewed expert, “the hardware-software system should enable creation of healthy 3D food products that are appealing to the consumers, while offering only the necessary customization options to not create a cognitive overload”.

#### 3.1.2. Use-Cases for 3D Food Printing

One of the interviewed experts from the industry emphasized that “3D food printing has a lot of potential, but capturing monetary value depends on where and how it’s utilized. Without an innovative use-case, 3D food printers may end-up like the bread machines from the 1990s”. According to another expert, “commercial high-volume 3D food printing platforms could be realistic within the next couple of years if there are attractive enough use-cases to kick-start the development work.” Table 4 lists various use-cases identified during the expert interviews. Some novel use-cases such as 3D food printing vending machines would require automation and integration of various functional elements. For example, there might be a need for the integration of functions such as intermediate storage of 3D printed food products in the 3D printer system. Automation of such systems could be carried out utilizing solutions available on the shelf. Furthermore, it seemed feasible to compile all the required functional elements into a working prototype, once the elements are available as optimized (which was not the state-of-the-art during the study).

#### 3.1.3. Maturity Level of 3D Food Printing Technology for Industrial Applications

A second set of interviews pointed out that extrusion-based 3D food printing systems have the most potential in terms of providing functional benefits to consumers. Material jetting in the context of 3D food printing could be only viewed as a finishing technique for customized products. Moreover, experts predicted that laser sintering and binder jetting techniques should remain in niche, since they are not yet applicable in producing nutritious food products. Currently available soft-material extrusion systems are found to be mature enough for industrial scaling. The paste could be made from a single ingredient or a mixture of ingredients, taking into account the personal dietary needs and preferences. Additionally, multiple-nozzle solutions could be employed to print food products with various ingredients separated. The case-specific R&D should focus on optimizing the rheology of ingredients, printing parameters, and possible post-processing (e.g., cooking, oven drying, and freezing). While there are no technological barriers associated with post processing, experts noted two important factors that must be taken into account. Firstly, the shape and size of the 3D printed food product could change during post-processing. This need to be considered while designing its 3D model. Secondly, similar to traditional food preparation, there is a close linkage between the processing method and the recipe used. Some ingredients might require a very specific way of post-processing in order to be palatable.

### 3.2. Evaluation Phase: Expert Survey and Consumer Focus Groups

#### 3.2.1. Survey Sample

Of the 85 surveys sent, 50 responses were received (sample size n = 50, with a response rate of 58.8%) from a sample population of several thousand. This implied a margin of error of 13.9%, considering a confidence interval of 95% and a response distribution of 50%. Here, the margin of error is defined as: Margin of error, e = z × (σ/√n); where n is the sample size, σ is the population standard deviation, and z is the z-score consistent with the desired confidence interval (for a confidence interval of 95%, z = 1.96). As shown in Table 5, majority of the responses were obtained from Finland (62%). There were also respondents from seven other European countries and five non-European countries, contributing to a global outlook in the quantitative study. 72% of the respondents were employed in academia or research organizations, whereas the remaining respondents represented different sectors associated with the 3D food printing value chain. Primary expertise of 92% of the respondents was amongst engineering/technology, food science, and business/management.

#### 3.2.2. Key Attributes of a 3D Food Printing Platform

**Figure 4:**
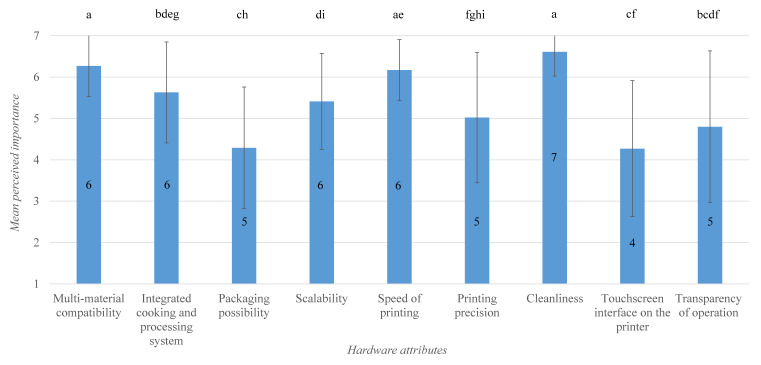
Bar plot of means for perceived importance of 3D food printer hardware attributes. Error indicates the standard deviation, and the number inside the bars indicates median. Bars sharing the same letter are not significantly different according to Wilcoxon signed rank test.

The Friedman test pointed out statistically significant difference(s) in respondents’ evaluation of hardware attributes, χ2(8) = 109.434, *p* = 0.000. Post hoc analysis utilizing Wilcoxon signed-rank test traced significant differences to 21 attribute pairs. Means and medians for the perceived importance of hardware attributes are represented in Figure 4. Here, the bars that do not share the same letters are significantly different according to the Wilcoxon signed rank test. For instance, there is a significant difference between the perceived importance of multi-material compatibility and integrated cooking/processing system (*Z* = −3.39, *p* = 0.001), and no significant difference between the perceived importance of multi-material compatibility and cleanliness (*Z* = −2.05, *p* = 0.040). Overall, experts perceived cleanliness to be the most important hardware attribute of a 3D food printer system (median perceived importance of 7) followed by multi-material compatibility, speed, integrated cooking/processing system, and scalability (median perceived importance of 6 each). While transparency, packaging possibility, and touchscreen interface were rated lower compared to other attributes (median perceived importance of 5, 5, and 4, respectively), they could still be considered as somewhat important (mean perceived importance higher than the mid-value of Likert scale).

**Figure 5:**
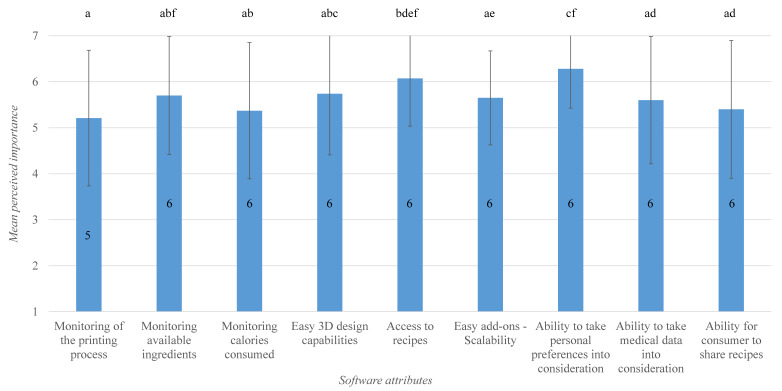
Bar plot of means for perceived importance of 3D food printer software attributes. Error indicates the standard deviation, and the number inside the bars indicates median. Bars sharing the same letter are not significantly different according to Wilcoxon signed rank test.

Friedman test pointed out statistically significant difference(s) in the respondents’ evaluation of software attributes, χ2(8) = 29.583, *p* = 0.000. Means and medians for the perceived importance of software attributes, as well as the six identified significant differences (via Wilcoxon signed-rank test) are represented in Figure 5. The test statistics showed a significant difference between the perceived importance of monitoring the 3D food printing process and access to recipes (*Z* = −3.23, *p* = 0.001). Additionally, how the experts perceived the importance of the ability to accommodate personal preferences (median perceived importance of 6) is significantly different from that of monitoring of printing process, monitoring calories, easy add-ons, ability to utilize medical data, and recipe sharing possibility (median perceived importance of 5, 6, 6, 6, and 6, respectively). In other words, while the experts considered all the identified software attributes to be fairly important, ability to take personal preferences into consideration and access to recipes were rated slightly higher than monitoring 3D food printing process, monitoring calories, easy add-ons, ability to utilize medical data, and recipe sharing possibility.

#### 3.2.3. Potential Use-Cases for 3D Food Printing

**Figure 6:**
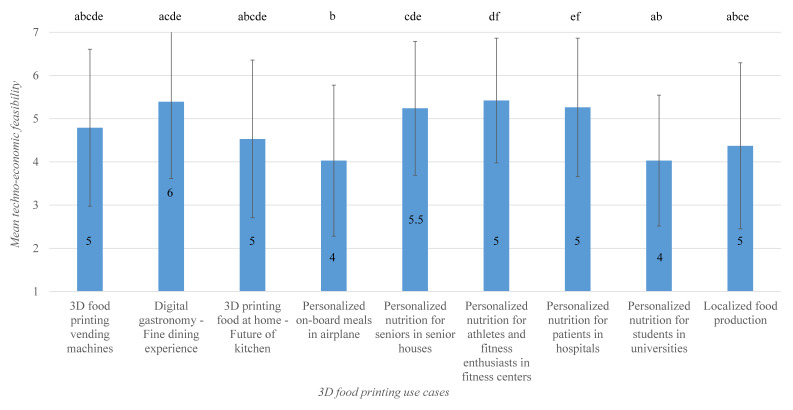
Bar plot of means for techno-economic feasibility of identified 3D food printing use cases. Error indicates the standard deviation, and the number inside the bars indicates median. Bars sharing the same letter are not significantly different according to Wilcoxon signed rank test.

The Friedman test conducted on the survey data related to the techno-economic feasibility of use-cases pointed out statistically significant difference(s), χ2(8) = 52.426, *p* = 0.000. The Wilcoxon signed-rank test that followed traced significant differences to eight use case pairs. Means and medians for techno-economic feasibility of 3D food printing use cases are as represented in Figure 6. Digital gastronomy/fine dining use case received a very high techno-economic feasibility rating from the experts (median techno-economic feasibility rating of 6). Similarly, the uses cases associated with implementation of 3D food printing in semi-public spaces, namely in fitness centers, hospitals, and senior homes received high techno-economic feasibility rating (median techno-economic feasibility rating of 5, 5, and 5.5, respectively). Interestingly, the use case—personalized nutrition for students in universities (median techno-economic feasibility rating of 4), which in practice is quite similar to other personalized nutrition use cases, obtained a comparatively lower techno-economic feasibility rating. It should also be noted that the novel use case of 3D food printing vending machines (median techno-economic feasibility rating of 5) was not rated significantly different from rest of the identified use cases in terms of techno-economically feasibility.

**Figure 7:**
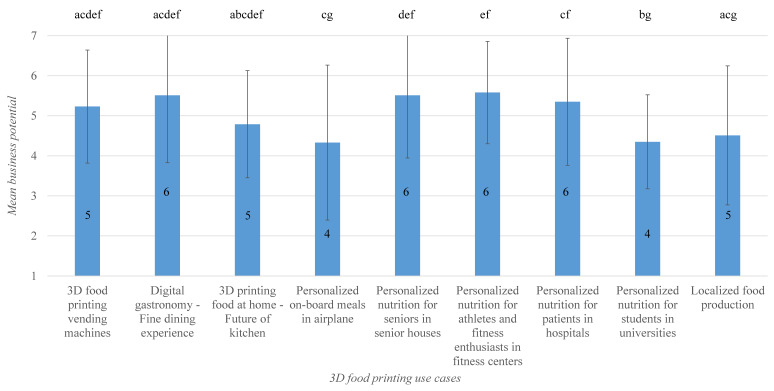
Bar plot of means for business potential of identified 3D food printing use cases. Error indicates the standard deviation, and the number inside the bars indicates median. Bars sharing the same letter are not significantly different according to Wilcoxon signed rank test.

The Friedman test conducted on the data related to the business potential of use-cases also pointed out statistically significant difference(s), χ2(8) = 50.157, *p* = 0.000. After post hoc analysis by means of Wilcoxon signed-rank test, nine use case pairs showed significant differences in terms of business potential. The means and medians for the business potential ratings of identified use cases are presented in Figure 7. Digital gastronomy/fine dining use case and the personalized nutrition use cases aimed at seniors, fitness enthusiasts, and patients in hospitals were found to have fairly high business potential (median business potential rating of 6 each). The use case of 3D food printing vending machines received a median and mean business potential rating of 5 and 5.23, respectively, and was not rated significantly different from other use cases except from the personalized nutrition use case targeted at university students (median business potential rating of 4). Here, the latter received a lower rating that is statistically significant (*Z* = −3.19, *p* = 0.001).

#### 3.2.4. Consumer Desirability of 3D Food Printing: Focus Groups

Consumer attitudes towards 3D food printing in the two countries (Finland and Belgium) were similar, and hence the findings of focus groups are presented together. Consumers were initially unfamiliar with the concept of 3D food printing, and the spontaneous reactions to the technology were mostly in negative terms. Most of the reactions were related to the name of the technology, which was considered very technical and unnatural. Their perceptions turned more positive after detailed introduction to the concept. While personalized nutrition potential and the convenience aspect of the technology were well recognized by the consumers, the design aspect of 3D printing was considered less important. There were several qualms regarding the quality and safety of 3D printed food and usability of the technology. Additionally, concerns related to eating experiences and food culture (e.g., loss of social aspect related to eating) were often mentioned. Overall, participants were interested in functional services for personalized nutrition rather than especial eating experiences.

The concepts of 3D food printing related to digital gastronomy, personalized snacks, and 3D food printing vending machines were presented to the focus groups in order to stimulate further development of ideas. Out of the three concepts, personalized snacks at fitness centers and 3D food printing vending machines in public spaces were found to be desirable to consumers. The former was more attractive, as it has clear purpose and a target group with real need. This also made the use case more realistic in consumers’ eyes. With regard to the 3D food printing vending machines in public spaces concept, consumers expressed doubts regarding the quality of food, type of food (e.g., whether it is warm or cold), and usability (e.g., will there be queues in metro stations if many people go to pick their order when metro arrives?). The digital gastronomy concept was rejected by consumers. It was not found to fit with the association of fine dining, i.e., the consumers expect the chef to prepare the food instead of the machine.

### 3.3. Development Phase: Business Modelling

Table 6 lists the design criteria that were defined to guide the business modelling workshops. The first four criteria corresponded to the four dimensions of business model, and the fifth was an additional criterion regarding overall sustainability. The first criterion made the link between business model creation and consumers’ attitude towards 3D food printing. This together with the second criterion helped in filtering out any use cases arising just from the fact that something could be 3D printed. The third criterion stated that the generated business models must be realizable within the next five years. In practice, this meant that components required to build 3D food printer systems must be commercially available presently so that any R&D required should be mainly related to optimizing the ingredients, recipes, and process parameters. The fourth criterion related to profit mechanism helped in filtering out any business models that focused on market creation by sinking the prices in a non-sustainable manner. The additional criterion helped in aligning the business model with the principles of sustainability, covering its environmental, social, and economic aspects.

Table 7 list three business models that were selected for further development after the first workshop with experts. The initial aim was to create manufacturer-driven, retailer-driven, and consumer-driven business models that are defined according to the physical location of 3D food printer system. However, the consumer-driven alternative was omitted because it was not found appealing by the consumer focus groups. The experts also did not foresee business potential in domestic appliances based on 3D food printing within the next five years. After evaluating and refining the three selected business models at the second workshop, their potential value chains were sketched.

#### 3.3.1. Customized Design Chocolates

Chocolate is one of the more common ingredients studied and optimized for 3D food printing [40,41,42]. 3D printing of customized design chocolates was selected as the first business model, since it could be realized presently utilizing the existing 3D food printer systems. In principle, the printing could take place at a manufacturer or at a retailer, e.g., a cafeteria. However, since high quality chocolate printing is not a simple task (as it is sensitive to the accuracy of recipe and printing parameters), the decision was made to build the business model around a chocolate manufacturer, i.e., a manufacturer-driven business model. The value proposition of chocolate manufacturer (i.e., 3D printed chocolate service provider) is to offer 3D printed design chocolates to customers who need a special eating experience as part of a small family party or a larger corporate event. There are two target customer groups for this value proposition: The first one is customers organizing a party, and the second one is restaurants and catering service providers organizing special business events.

**Figure 8:**
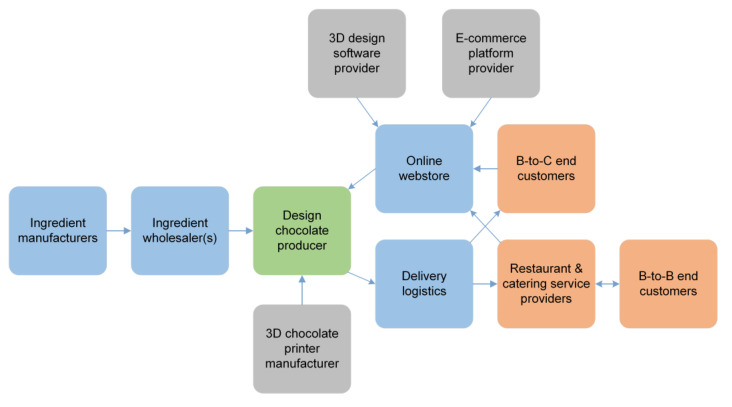
Value chain of the business model—Customized design chocolates.

The chocolate design and other customizations would be made by the customer using the online software platform/webstore of the 3D printed chocolate service provider. After receiving the online order from the customer, the 3D printed chocolate service provider produces (3D-prints) the customized design chocolates and sends them directly to the customer. The required internal key resources include 3D printers suitable for chocolate printing as well as equipment for flavoring and cartridge filling. Internal core competences and activities consist of chocolate making, cartridge filling, print-design capability, and process management. External key competences required from partners consist of e-commerce platform, delivery logistics, 3D chocolate printer manufacturer (and maintenance service, if necessary), software development, providing of high-quality ingredients for chocolate making, and marketing and branding competence. The value chain of this business model from the standpoint of 3D printed chocolate service provider could be illustrated as in Figure 8.

Earning logic in this business model could be based on price per printed chocolate with bulk order discounts. As the competition is not against the mass production of chocolates, the offering could be premium prized. Customized design chocolates is clearly a business model for niche markets and, accordingly, represents a small business. Investment costs and risks related to the business model are low or moderate. The business model supports sustainability because the printing process will take place only according to the orders received. This means that there will be no non-marketable items in stock at any given time. Furthermore, the shorter value chain provides more value for each actor present in the chain and makes it easy to trace the ingredients and final products. Another positive aspect related to this business model is that all the required functional elements are either currently available or only require slight modifications.

#### 3.3.2. Personalized Snacks

According to the focus groups and survey results, the highest business potential for 3D food printing could be in places where people with needs for personalized nutrition gather, like at fitness centers and sport halls. With this in mind, use cases related to personalized 3D printed snacks at fitness centers and other semi-public spaces like sport halls and offices were taken in for further development. The use cases could either facilitate a manufacturer-driven business model, in which the 3D printing will take place centralized at a manufacturer by order, or a retailer-driven model, in which the 3D printing will take place at the point of use. Only the latter possibility was considered further. In order to bring-in novelty and diversity, a vending machine approach was chosen for the delivery of 3D printed personalized snacks to customers. The business model was designed from the standpoint of a vending machine operator.

The value proposition of vending machine operator is to offer personalized healthy snacks, prepared by an order in a vending machine, using 3D food printing technology and possible post processing operations. This would in turn foster healthy and vitalizing eating habits among the target customer groups. Ordering takes place using a mobile application that takes into account the personal dietary requirements and other consumer preferences. The vending machine offers 1–3 base pastes, to which additional protein, carbohydrates, vitamins and minerals, as well as extra flavors could be added before printing for attaining consumer-desired personalization. In order to shorten the waiting time, there will be a possibility to pre-order the snack that will subsequently be printed and stored within the vending machine for customer pick-up. Earning logic could be based on a subscription model (value card, monthly subscription, etc.), instead of payment per order.

**Figure 9:**
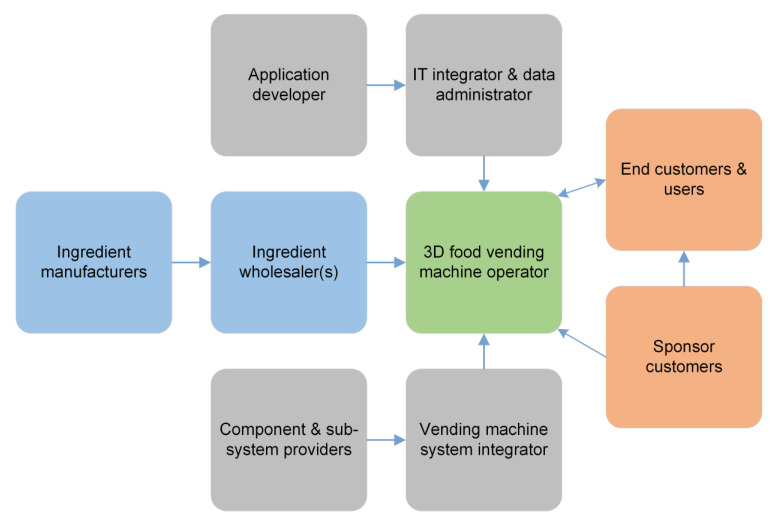
Value chain of business model—Personalized snacks.

Direct target customers in this business model are athletes/recreational athletes as well as office workers. Indirectly, the employers and the parents of young athletes are also target groups of this business model. The most important internal key resources of the vending machine operator are the actual vending machines, 3D food printers, ingredient cartridges, an automatic mixer for personalized printing-paste creation, an oven for post processing, locked storages for printed snacks, and the basic digital vending machine automation. External key competence and resources required from partners consists of the 3D food printing vending machine system (partnering with a system integrator, who designs and assembles the components and sub-systems of the vending machine), e-commerce platform for subscriptions, application software development and support for the user interface, data administration, and providers of ingredients. The value chain of this business model could be as presented in Figure 9.

The business model, personalized snacks, represents a business opportunity that could be characterized by a word pair of high risk—high reward. While the markets for personalized snacks are quite large, the transformation of this business model into a real business would require 3D food printing vending machines to truly fulfil the needs and expectations of users (customers). Additionally, the price of the snacks will matter and must be comparable to traditional snack offerings. This could be challenging in the start-up phase of the business when the high investment costs of equipment are to be amortized. In addition to the aforementioned, there are also technological risks. 3D food printing vending machine is a completely new machine concept. Although the technology itself exists and is mature enough at component level, many design and engineering R&D efforts are required before a system level equipment would be available. While attaining the first functional prototype might not be very challenging, it would still be a long route from its inception to an easy-to-use commercial system that is also cost-effective.

#### 3.3.3. 3D Food Printing in Kitchen Department of Hospitals

The third business model, 3D food printing in kitchen department of hospitals, was developed based on the use case of personalized meals at hospitals. It was created around the concept of a digital kitchen. The focal firm in the business model is a catering service provider for hospitals that has its own kitchen department for food preparation. Target customers are private and public hospitals as well as hospital districts, where patients with specific food needs are the end users of the service. The value proposition of the model is a personalized meal service to promote patient recovery. The smart, digital kitchen department system produces and delivers personalized meals in large volumes required to serve all patients at mealtimes.

**Figure 10:**
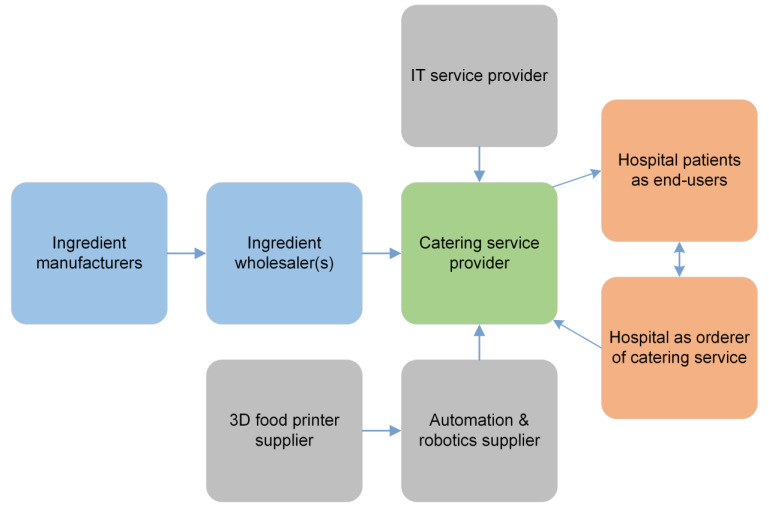
Value chain of business model—3D food printing in kitchen department of hospitals.

The model refers to a semi-automated process that involves co-working of kitchen personnel with different kind of robotic systems, including 3D food printers. The digital kitchen has an interface to the patient IT system of the hospital, where 3D food printers receive input data for personalization. Although the process is partially digitalized, the internal core competence of a catering service provider still comes from basic kitchen department operations. These are just supplemented by automation and robotics. External key competence and resources required from partners consist of the design and delivery of robotics and automation system, their maintenance, IT system development, and operations needed to run the digital kitchen. The value chain associated with this business model could look like that in Figure 10.

This model would support sustainability in two significant ways. Firstly, it could reduce food waste as meals are produced according to personal needs. Secondly, it could offer remarkable social benefits if it shortens the recovery time of patients. It is highly likely that automation and robotics will come into kitchen departments sooner or later. While the technology is mature enough at component level, more R&D and piloting is still required at process and system levels. Nor will the investment costs for digital kitchen be on a smaller scale. Whether or not 3D food printers will be part of this digital kitchen will largely depend on the capability of the system to produce large volumes of personalized meals, or parts of meals, in a short period of time.

## 4. Discussion

3D food printing has gained a lot of interest in the recent past and is considered as a field with high expectations. The aim of this study was to update the outlook of 3D food printing beyond its general anticipation by addressing the identified knowledge gaps in the field. The results generated should help the industry and academia to channel their research and developmental activities to best exploit the functional value of 3D food printing. Firstly, the results provided an update on the maturity level of the technology for industrial-scale utilization. Additionally, the key attributes of 3D food printer hardware-software system were put together. Secondly, the study discerned prospective use cases for 3D food printing that are techno-economically feasible for businesses to implement, and desirable to the consumers. Lastly, three business models were provided together with value chain sketches to aid various commercialization efforts.

The short feasibility study with experts pointed out that 3D food printing technology at component level is mature enough for industrial utilization. This means that individual aspects of the technology, such as the state of engineering in 3D food printing, ingredient portfolio, and post-processing options can presently be utilized on an incremental basis. One of the associated challenges, identified from the literature [14,15,16], is the matching of food ingredients with suitable 3D printing techniques. However, out of the techniques applicable in 3D printing food (i.e., material extrusion, selective laser sintering, hot air sintering, and liquid binding), material extrusion provides the most versatility in terms of applicable ingredients [8,15,43,44]. In agreement with this, experts considered extrusion-based techniques to have the most potential for industrial scaling.

Godoi et al. have categorized extrusion-based 3D food printing techniques into soft-material extrusion, melting extrusion, and hydrogel-forming extrusion [15]. The experts favored soft-material (paste) extrusion, and were keen on multi-material printing possibilities. The reason for this favoring could be due to the fact that such multi-material systems are already well developed [45] and are commercially available [46]. Additionally, the available literature pinpoints a variety of healthy and alternate base paste ingredients that could be used with soft-material extrusion [10,44]. However, the quality of the extruded food product depends on critical material parameters, such as rheological properties, particle size, mechanical properties, etc., as well as processing parameters like nozzle diameter, temperature, extrusion rate, solidification rate, etc. [15,20]. Regarding post-processing, experts raised concerns about shape retention and compatibility of traditional methods including baking and freeze-drying. The latest research addresses this challenge. Lille et al., for example, managed to significantly improve the shape retention of milk formulations by using rye flour as an additive [10].

Hardware and software attributes were evaluated by the survey participants for its importance, only on a generic level. Industry/academia must examine these attributes for its relevance in the context of the use case they have in hand. For example, a use case related to 3D chocolate printing would benefit more from having better printing precision rather than multi-material compatibility. Additionally, integrated cooking system is not a relevant hardware attribute for such a use case. Previous studies [21,22] focused on the specialty goods sector points out the significant role that an intuitive software platform plays in leveraging functional value of a technology like 3D printing. The attributes and customization parameters identified as part of this study could be utilized by 3D food printer manufacturers for developing systems that are connected, data-driven, collaborative and transparent.

Regarding survey results on the techno-economic feasibility and business potential of identified use cases, the ones related to personalized nutrition (of athletes, patients at hospitals, and seniors) were very well-received. However, the use case of personalized nutrition for students in universities was an exception. This could be due to the fact that athletes, patients, and senior citizens are more specific target groups compared to students, and hence the implementation process would be comparatively easier. The study results additions to the existing knowledge regarding personalized nutrition as an application area of 3D food printing [47,48,49]. Interestingly, the 3D food printing vending machine use case was not rated significantly different from personalized nutrition (of athletes, patients at hospitals, and seniors) use cases in terms of techno-economic feasibility. This, however, does not mean that 3D food vending is equally feasible as the latter use cases. The reason for this higher rating could be its novelty value, and that the survey participants might have had different perceptions regarding its implementation.

According to the literature, already taking consumer perspectives into account during the early development phase will have a positive impact on the market acceptance of novel food technologies [50]. The results of this study reflected the novelty of 3D food printing to consumers and highlighted the importance of introducing the technology in markets via services that are realistic, and with appropriate target groups. Focus group results indicated that consumers desire personalization and convenience over special eating experience. It is not advised to brand the technology as 3D food printing, as the term ‘printing’ drew adverse associations in consumers’ minds that are against the current food trends related to naturalness and healthiness. In general, the focus group results confirmed the finding of Mantihal et al., that consumer awareness of 3D food printing is still very limited [8]. 3D food printing enterprises should invest resources to address this issue, since consumers ultimately decide whether 3D food printing will make a commercial breakthrough or not.

The results from the exploratory phase and the evaluation phase were utilized in creating the three business models. Although the presented models would not be realized as such, they include elements that could be used when designing other models. It should be noted that only an upper-level business model sketching is carried out to answer how firms could create and capture value utilizing the identified use-cases. The next step would be to create machine design concepts for the selected models and to test them with end-users. Refined machine design concepts could then be translated to working prototypes.

## 5. Conclusions

This study investigated the techno-economic prospects as well as consumer desirability of 3D food printing utilizing a mixed methods approach. The data obtained from expert interviews were evaluated using an expert survey and consumer focus groups. The results provided perceptions on the key hardware-software attributes and use cases for 3D food printing. Both experts and consumers supported 3D food printer (hardware-software) platforms that could offer functional value in terms of health, nutrition, and convenience. The research results were translated into three business models for 3D food printing, namely, customized design chocolates, personalized snacks at semi-public spaces, and 3D food printing in kitchen department of hospitals. The first model could easily be implemented presently by small businesses around the niche, with minimal resources. However, the latter models involving the 3D printing of personalized meals/snacks for large consumer segments, would offer higher business prospects. Although no technological and scientific breakthroughs are necessary, the development efforts required to make these business models real and profitable should not be underestimated. The personalized snacks model incorporates the novel machine concept of 3D food vending, and therefore would demand (high) risk taking to start the development efforts. If such risks are taken and if those attempts are successful, 3D printed food could indeed become a part of our daily life. If not, 3D food printing might remain in niche with models such as customized design chocolate, which target small and specific segments.

## Figures and Tables

**Table 1:** Questions and the corresponding number of responses that were analyzed in SPSS.

Questions Analyzed in SPSS	Responses, N
Rate the attributes of 3D food printing hardware based on importance	41
Rate the attributes of 3D food printing software based on importance	43
Rate the 3D food printing use-cases based on business potential	43
Rate the 3D food printing use-cases based on techno-economic feasibility	38

**Table 2:** Key change drivers for 3D food printing and its characteristics.

Change Drivers	Characteristics
ICT revolution	Role of smart phones, automation possibilities
Centralized to on-demand food production	Resource optimization, convenience
Demand for customized food products	Personalized nutrition
Novel food ingredients	Healthy and sustainable alternatives
Recent boom in food-tech market	Dynamic application areas

**Table 3:** Hardware and software attributes of 3D food printer system identified during the exploratory phase.

Hardware Attributes	Software Attributes
Cleanliness and safety	Ability to take-in personal preferences
Multi-material compatibility	Ability to utilize medical data
Scalability	Access to recipes
Transparency of operation	Monitoring of available ingredients
Integrated cooking/processing system	Monitoring of printing process
Packaging possibility	Monitoring calories consumed
Printing precision	Easy 3D design capabilities
Touchscreen interface	Easy add-ons (scalability)
Speed of 3D printing	Recipe sharing possibilities

**Table 4:** Use-cases for 3D food printing identified during the exploratory phase.

1. 3D food vending machines—Vending machines integrated with 3D food printers
2. 3D food printing in fine dining—Digital gastronomy
3. 3D food printing in home kitchens
4. Personalized on-board meals in airplanes
5. Personalized nutrition at senior homes
6. Personalized nutrition at fitness centers in the form of 3D printed snacks
7. Personalized nutrition for patients at hospitals
8. Personalized nutrition for students at universities and schools
9. Localized food production—3D food printing on wheels

**Table 5:** Survey demographics.

Demographic	Number of Responses	Share (%)
Country where the respondents work:		
Finland	31	62.0
Other European countries	13	26.0
Non-European countries	6	12.0
Primary expertise of the respondents:		
Engineering/Technology	18	36.0
Food related research	15	30.0
Business/Management	13	26.0
Others	4	8.0
Sector of occupation:		
Research, university, or education	36	72.0
3D food printer developer/manufacturer	3	6.0
Service provider (software, hardware, design agency, etc.)	3	6.0
Food ingredients producer or food processor	3	6.0
Food distributer (retailer, restaurant/bakery, catering, etc.)	2	4.0
Others	3	6.0

**Table 6:** Design criteria defined and the corresponding business model dimensions.

Design Criteria	Dimension
1. There must be a clearly identified customer need for the value proposition.	Market domain
2. The value proposition must be somehow superior to the current offerings.	Value Proposition
3. The business model must be realizable within the next 5 years (by 2024).	Technical domain
4. Targeted business must be profitable already in the short run.	Profit mechanism
5. Business models should be aligned with principles of sustainability	Sustainability

**Table 7:** Three business models generated in the development phase.

1. Customized design chocolates
2. Personalized snacks
3. 3D food printing in kitchen department of hospitals

## Appendix A. Framework Utilized for the First Set of Interviews with Experts

*Duration: 30 min to 45 min*

Warm-up and introduction:

- Introducing myself and the research—very briefly.
- Please tell about your education/research background.
- Company/project background.
- What is your general opinion about 3D printing of food?
  ○ What comes to your mind first when you think about it?

3D food printing (general):

- What in your opinion would be the biggest value of 3D food printing?
  ○ For consumers?
  ○ For food industry/other businesses?
- What in your opinion is the single biggest challenge in 3D food printing? Why?
  ○ With regards to technology feasibility.
  ○ With regards to economic viability.
  ○ With regards to consumer desirability.
- Do you see a potential in the 3D food printing technology for disrupting the food value chain? How would it help in doing so?
- Which is the most suitable technology for 3D food printing? Why?
- How important is pre-processing and post-processing in case of 3D food printing? Do you see a value in integrating a cooking/processing system to 3D food printer?

Future of food:

- What are the key trends and uncertainties in our food system?
- Other than the technology push, what are the factors that might impact the food industries?
  ○ Does it complement technology innovations like 3D food printing? How?
- In your opinion, which is the food ingredient of the future?

ICT innovation and rise of prosumers:

- What is your take on the emerging online platforms and its role in allowing consumers to be a part of the production process (Prosumers)?
- Do you think that the ICT developments and tech-innovations like 3D printing allow consumers to take part in the value creation process with ease? Or are there still some limitations?
- What are the characteristics of an ideal prosumer platform (hardware-software system)?
- What is the most important parameter for food customization in your opinion?
- What does the prosumer want to customize in food?

Wrap-up:

- How would you envision the future of food production? Does 3D food printing play a role in it?
- Most probable scenario for 3D food printing in 2025?
- Are you willing to answer a survey that I will be preparing to finalize the collected data?

## Appendix B. Focus Group Protocol *(Only Part 2 and Part 4 Are Utilized in This Paper)*

*Part 1: Future of food and food consumption*

Future of food consumption (10 years from today):

- What do you think food and food consumption will be like 10 years from now?
  ○ Will it be different and how?
  ○ What kind of needs related to food and the consumption of food might there be in the future?
  ○ What do you expect from the food in future?
    ▪ What is important? Why?
    ▪ Do you have some concerns when you think about food and future? Why?
    ▪ Do you have some hopes for the future in terms of food and food consumption?

*Part 2: 3D food printing*

Spontaneous reactions to 3D food printing:

- What comes to mind for you when 3D Food printing is mentioned? Do you know what it means? Does it evoke any emotions?
- Have you heard about it before? Where?

Perceptions, beliefs and fears (after detailed introduction of the concept):

- What comes to mind for you based on this description of 3D Food printing?
- What are the positives of this approach to creating meals and snacks?
- Do you have any concerns related to it?
- Does the idea of 3D food printing presented in the text make any sense?

Potential ways of using:

- Would you use 3D Food printer?
- How would you use it?
- Where would you use it?
- Could someone else be interested in 3D Food printing? How and under which circumstances they would use it?

*Part 3: Exploration of new creative ideas for 3D Food printing*

Brain storming in small groups to create new ideas for 3D Food printing:

There is a so-called user profile for each small group. In the user profile, there is a description of a fictional person. Your task is to come up with some ideas on how 3D printing could be used to create food solutions that support this person. You should describe the following characters of the solution you create:

- What are the key features of the solution? What is needed to fulfil person’s goal?
- What is the end product like?
- Where the product is produced and how it is delivered?
- What is the price?
- How it should be promoted?

*Part 4: Evaluation of 3D food printing concepts*

Participants’ perceptions of the concepts and further development ideas:

- In general, what do you think about the idea? How does it make you feel? Is it interesting or boring?
- What benefits do you associate with the concept?
- What downsides do you associate with the concept?
- Do you have some concerns related to the concept?
- Would you add something to the concept or take something away?
- Could the idea presented in the concept be applied in some other context? In which and for what reason?
- Would you be interested in using Digital gastronomy/Personalized snacks at fitness centers/3D food printing vending machines in public spaces as described in the concept?
- Who else could be interested in Digital gastronomy/Personalized snacks at fitness centers/3D food printing vending machines in public spaces? What would be the reasons for interest?
- In your opinion, would Digital gastronomy/Personalized snacks at fitness centers/3D food printing vending machines in public spaces be a momentary fad or would it last?
- Could the idea be applied in some other context than Fine dining/Personalized snacks at fitness centers/3D food printing vending machines in public spaces?
